# EGCG protects the mouse brain against cerebral ischemia/reperfusion injury by suppressing autophagy *via* the AKT/AMPK/mTOR phosphorylation pathway

**DOI:** 10.3389/fphar.2022.921394

**Published:** 2022-09-06

**Authors:** Li Wang, Maosha Dai, Yangyang Ge, Jiayi Chen, Chenchen Wang, Chengye Yao, Yun Lin

**Affiliations:** ^1^ Department of Anesthesiology, Union Hospital, Tongji Medical College, Huazhong University of Science and Technology, Wuhan, China; ^2^ Department of Neurology, Union Hospital, Tongji Medical College, Huazhong University of Science and Technology, Wuhan, China

**Keywords:** (−)-epigallocatechin-3-gallate (EGCG), autophagy, cerebral ischemia/reperfusion injury (CIRI), middle cerebral artery occlusion/reperfusion (MCAO/R), oxygen-glucose deprivation/reoxygenation (OGD/R), HT22 cell, AKT/AMPK/mTOR pathway

## Abstract

Stroke remains one of the leading reasons of mortality and physical disability worldwide. The treatment of cerebral ischemic stroke faces challenges, partly due to a lack of effective treatments. In this study, we demonstrated that autophagy was stimulated by transient middle cerebral artery occlusion/reperfusion (MCAO/R) and oxygen-glucose deprivation/reoxygenation (OGD/R). Treatment with (−)-epigallocatechin-3-gallate (EGCG), a bioactive ingredient in green tea, was able to mitigate cerebral ischemia/reperfusion injury (CIRI), given the evidence that EGCG administration could reduce the infarct volume and protect poststroke neuronal loss in MCAO/R mice *in vivo* and attenuate cell loss in OGD/R-challenged HT22 cells *in vitro* through suppressing autophagy activity. Mechanistically, EGCG inhibited autophagy *via* modulating the AKT/AMPK/mTOR phosphorylation pathway both *in vivo* and *in vitro* models of stroke, which was further confirmed by the results that the administration of GSK690693, an AKT/AMPK inhibitor, and rapamycin, an inhibitor of mTOR, reversed aforementioned changes in autophagy and AKT/AMPK/mTOR signaling pathway. Overall, the application of EGCG relieved CIRI by suppressing autophagy *via* the AKT/AMPK/mTOR phosphorylation pathway.

## Introduction

The world is facing an epidemic of stroke. Of all strokes, cerebral ischemic stroke (CIS) accounts for almost 87% (Virani et al., 2021) with a high risk of mortality and severe long-term disability, placing an increasing economic burden on family and society (Feigin et al., 2018). Tissue plasminogen activator (tPA) is the only supported pharmacological thrombolytic medicine because of its ability to recanalize arteries and improve clinical outcomes (National Institute of Neurological Disorders and Stroke rt-PA Stroke Study Group, 1995). Restoration of the blood supply into the ischemic stroke region may also induce cerebral ischemia/reperfusion injury (CIRI) (Lim et al., 2021). Moreover, only a shortage of patients with CIS benefits from tPA treatment due to the narrow therapeutic time window, tendency to transform into a hemorrhage, and other side effects (Bansal et al., 2013; Yeo et al., 2013; Bhaskar et al., 2018). Thus, there is a compelling need to explore more effective therapies to promote ischemic tissue recovery and ameliorate patient prognosis after stroke.

In the pathophysiological process of stroke, several types of neuronal death can be triggered, involving autophagy, apoptosis, necrosis, necroptosis, and pyroptosis (He et al., 2020). Among them, autophagy has been extensively studied in CIRI over the past decades. As a regulated intracellular degradation process, autophagy is demonstrated to maintain normal cellular functions and cellular homeostasis and is involved in some neurodegenerative diseases by degrading and recycling dysfunctional or damaged organelles or proteins (Srivastava et al., 2017; Wang P. et al., 2018; Zhang et al., 2019). Basal autophagic activity exists in cells under physiological conditions, while the process turns more active induced by various stress events including CIRI and plays complex roles (Wirawan et al., 2012). Some studies demonstrated that the activation of autophagy ameliorated CIRI (Sun et al., 2020b; Xu et al., 2020; Zhang B. et al., 2020; Chen et al., 2021; Jin et al., 2021; Yihao et al., 2021; Zha et al., 2021), while more research supported that the inhibition of autophagy activation exerted a neuroprotective effect in brain stroke (Sun et al., 2020a; Zhang et al., 2020b; Li and Huang, 2020; Mei et al., 2020; Shi et al., 2020; Wang L. et al., 2020; Gu et al., 2021; Liu N. et al., 2021; Shao et al., 2021; Wang C. et al., 2021; Xu et al., 2021; Yao et al., 2021; Zhang et al., 2021; Zhao et al., 2021). Obviously, the controversial dual role of the induction of autophagy in the ischemic brain is still a hot topic in research and remains to be further investigated.

There is a growing focus on treating stroke with natural medicines (Jahan et al., 2018), which could assist the body to regain and retain internal balance by providing external stimuli (Tao et al., 2020). Green tea is a celebrated traditional herbal medicine and is popular in the world, with many potentially beneficial effects on human health for its anti-cancer and anti-inflammatory properties (Saeed et al., 2017; Musial et al., 2020). (−)-Epigallocatechin-3-gallate (EGCG) is the most abundant and active polyphenol, accounting for 50%–80% of all catechins, and is believed to make a major contribution to the various benefits of green tea (Prasanth et al., 2019). Accumulating evidence suggested that EGCG conferred a neuroprotective effect in the acute and delayed states of stroke (Han et al., 2014; Zhang et al., 2015; You, 2016; Bai et al., 2017; Wang and You, 2017; Zhang et al., 2017; Park et al., 2020). The effect of EGCG on autophagy has been described in myocardial ischemia/reperfusion injury (MIRI) (Xuan and Jian, 2016; Zhang C. et al., 2020; Liu P. et al., 2021) and some other diseases (Musial et al., 2021; Du et al., 2022; Wang et al., 2022). However, it is not clear whether EGCG ameliorates CIRI through modulating autophagy, which needs to be elucidated. Moreover, there is a long way to go to translate the success of EGCG in animal research to humans. Thus, more consideration should be taken to further elucidate the detailed mechanisms by which EGCG exerts a neuroprotective effect on stroke.

Herein, we investigated the potential effect of EGCG on neuronal injury and autophagy activation. The present results indicated that EGCG exerted a protective effect on ischemic injury through the suppression of autophagy, which was induced by MCAO/R models of mice *in vivo* and OGD/R models of HT22 cells *in vitro*. In addition, the AKT/AMPK/mTOR phosphorylation pathway might be related to the inhibitory influence of EGCG on autophagy activation, which remains to be elucidated.

## Materials and methods

### Reagents

EGCG, anti-LC3B antibody, and anti-NeuN antibody were purchased from Sigma-Aldrich (USA). Donkey anti-mouse IgG secondary antibody was obtained from Life Technologies (Thermo Fisher Scientific, United States). Phosphor-AKT (p-AKT), AKT, p-AMPK, AMPK, p-mTOR, mTOR, beclin1, β-actin, and GAPDH were obtained from ABclonal Technology Co., Ltd. (China). P62/SQSTM1 polyclonal antibody was obtained from Proteintech (China). Horseradish peroxidase (HRP)-conjugated secondary antibody was obtained from Cell Signaling Technology (United States). GSK690693, rapamycin, and LY294002 were obtained from MedChemExpress (United States). Fetal bovine serum (FBS) and 4′,6-diamidino-2-phenylindole (DAPI) were purchased from Gibco (Thermo Fisher Scientific, United States). The antifade mounting medium was obtained from Solarbio (China). RIPA lysis buffer was obtained from Beyotime (China). Protease and phosphatase inhibitor cocktails and NcmBlot blocking buffer were obtained from New Cell and Molecular Biotech (China). BCA protein assay kit was obtained from CWBIO (China). Cell Counting Kit-8 (CCK8) and ECL chemiluminescent HRP substrate A&B were obtained from Antgene (China). All other reagents, unless stated otherwise, were obtained from Biosharp (China).

### Experimental animals and treatments

Adult male wild-type (WT) C57BL/6 mice were obtained from Beijing Weitong Lihua Experimental Animal Technical Co., Ltd., China. Throughout the experiment, the mice were kept in the SPF conditions in the Laboratory Animal Center, having food and water freely and avoiding sound and light stimulation. MCAO/R model was established as our previous protocols (Ge et al., 2022). After anesthetizing intraperitoneally (i.p.) in mice (8–12 weeks old, 23–26 g), a 6–0 silicone-coated nylon monofilament was used to obstruct the origin of the MCA to induce focal cerebral ischemia and establish MCAO model. Reperfusion was achieved by gently withdrawing the monofilament after 1 h of occlusion. Body temperature was sustained at 37 ± 0.5°C with an electric blanket until the mice had recovered from surgery. After waking from anesthesia, mice were housed in their cages with conditions as before. Sham group mice underwent the same surgical procedures as MCAO/R models without the obstruction of MCA. The experiment was approved by the Institutional Animal Care and Use Committee at Tongji Medical College, Huazhong University of Science and Technology.

Experimental groups were distributed randomly. Reagents were injected slowly (0.1 μl/min) into the right ventricle (1.5 mm laterally, 0.6 mm posteriorly, 3.1 mm deep from the anterior fontanelle). Mice were randomly divided into seven groups: 1) Sham group: mice received a surgical operation without MCAO/R; 2) MCAO/R group: mice received MCAO/R; 3) MCAO/R + vehicle group: mice were subjected to MCAO/R followed by vehicle (4 μl) injection 1 h later; 4) MCAO/R + EGCG group: mice were subjected to MCAO/R followed by EGCG (1 μg/μl, 4 μl) injection 1 h later; 5) vehicle + MCAO/R + EGCG group: the vehicle (4 μl) was injected 10 min before MCAO/R, followed by EGCG (1 μg/μl, 4 μl) injection 1 h later; 6) GSK690693 + MCAO/R + EGCG group: GSK690693 (10 μM, 4 μl) was injected 10 min before MCAO/R, followed by EGCG (1 μg/μl, 4 μl) injection 1 h later; and 7) rapamycin + MCAO/R + EGCG group: rapamycin (10 μM, 4 μl) was injected 10 min before MCAO/R, followed by EGCG (1 μg/μl, 4 μl) injection 1 h later.

### Cell culture and treatments

HT22 cells were cultured in Dulbecco’s modified Eagle’s medium (DMEM) with glucose, added with 10% FBS and 1% penicillin–streptomycin solution at 37°C in 95% air and 5% CO_2_. To simulate CIS *in vitro*, HT22 cells were challenged by OGD/R. In brief, HT22 cells were cultured in DMEM with no glucose and FBS at 37°C in 1% O_2_, 5% CO_2_, and 94% N_2_ for 12 h and then reoxygenated in the aforementioned normal growth environment. Vehicle and EGCG (20 μM) were added to HT22 cells at the time of reoxygenation and incubated for 2 and 4 h. LY294002 (25 μM), GSK690693 (25 μM), and rapamycin (25 μM) were added into HT22 cells at 0.5 h before the administration of EGCG and incubated for 2 h.

### 2, 3, 5-Triphenyltetrazolium chloride (TTC) staining

On post-surgery day (PSD) 1, infarct volume was assessed by TTC staining. Mice were anesthetized and then killed by the cervical dislocation method. The brains were dissected carefully and rapidly and refrigerated for 20 min at −20°C. After that, each brain was cut coronally into 2-mm-thick thin slices. And slices were put into a 6-well plate and immersed in 2% TTC for 15 min at 37°C. Gentle stirring ensured even staining exposure. Viable brain tissue was stained into deep red by TTC staining, while infarcted tissues kept the original pale color. Slices were fixed in 4% paraformaldehyde in phosphate buffer overnight and then recorded with pictures. An ImageJ analysis system (Version:2.1.0) was used to quantify the infarct volume and total brain volume. The cerebral infarct volume was calculated as the percentage of the infarcted tissue volume to the total brain tissue volume.

### Immunofluorescence staining

On PSD 1, total brain tissues were firstly perfused with saline solution, and then replaced the saline with 4% paraformaldehyde in phosphate buffer and continued perfusing. Afterward, the brains were dissected quickly and then fixed in 4% paraformaldehyde overnight at 4°C. Going through dehydration, transparency, waxing, and embedding successively, the fixed brains were then sliced into 4-μm-thick coronal slices. After dewaxed and antigen repaired at high temperature and high pressure, the sections were incubated in 10% donkey serum at room temperature for 20 min; next, the sections were incubated with primary antibodies, light chain 3 (LC3) antibody (1:200), and NeuN antibody (1:100), overnight at 4°C. After washing with phosphate-buffered saline (PBS) three times, slices were treated with donkey anti-mouse IgG secondary antibody (1:400) at room temperature in a dark place for 30 min, and next washed with PBS three times. DAPI was used to counterstain nuclei. The sections were finally observed and pictured under fluorescence photography and then analyzed by utilizing ImageJ software.

### Western blotting assay

The Western blotting assay was completed as our previous protocols (Ge et al., 2022). After the homogenization of the ischemic brain tissue, the BCA Protein Assay Kit was used to quantify the protein concentration of samples and then adjusted the protein concentration to 2 μg/μl with the RIPA lysis buffer. Proteins were analyzed by sodium dodecyl sulfate-polyacrylamide gel electrophoresis (SDS-PAGE) and turned onto PVDF membranes. After blocking with an NcmBlot blocking buffer for 20 min, the membranes are incubated with primary antibodies, phosphor-AKT (p-AKT) (1:1,000), AKT (1:1,000), p-AMPK (1:1,000), AMPK (1:1,000), p-mTOR (1:1,000), mTOR (1:1,000), p62 (1:1,000), LC3 (1:1,000), beclin1 (1:1,500) β-actin (1:5,000), and GAPDH (1:5,000) overnight at 4°C. After washing, the membranes were incubated with the corresponding horseradish peroxidase (HRP)-conjugated secondary antibody (1:5,000) for 1.5 h at room temperature. Chemiluminescence detection was carried out with ECL chemiluminescent HRP substrate A&B and captured through an imager machine. Band optical intensity was quantified with ImageJ software.

### Cell viability assay

The cell viability of HT22 cells was assessed with a cell counting kit (CCK-8) by the manufacturer’s protocols.

### Statistical analysis

Multiple comparisons were performed by a one-way analysis of variance (ANOVA) followed by Tukey’s multiple comparison tests for multiple comparisons (GraphPad Prism statistics software version 9.0.2, La Jolla, CA, United States). A *p*-value of <0.05 was considered statistically significant.

## Results

### EGCG mitigated CIRI in MCAO/R mice

**FIGURE 1:**
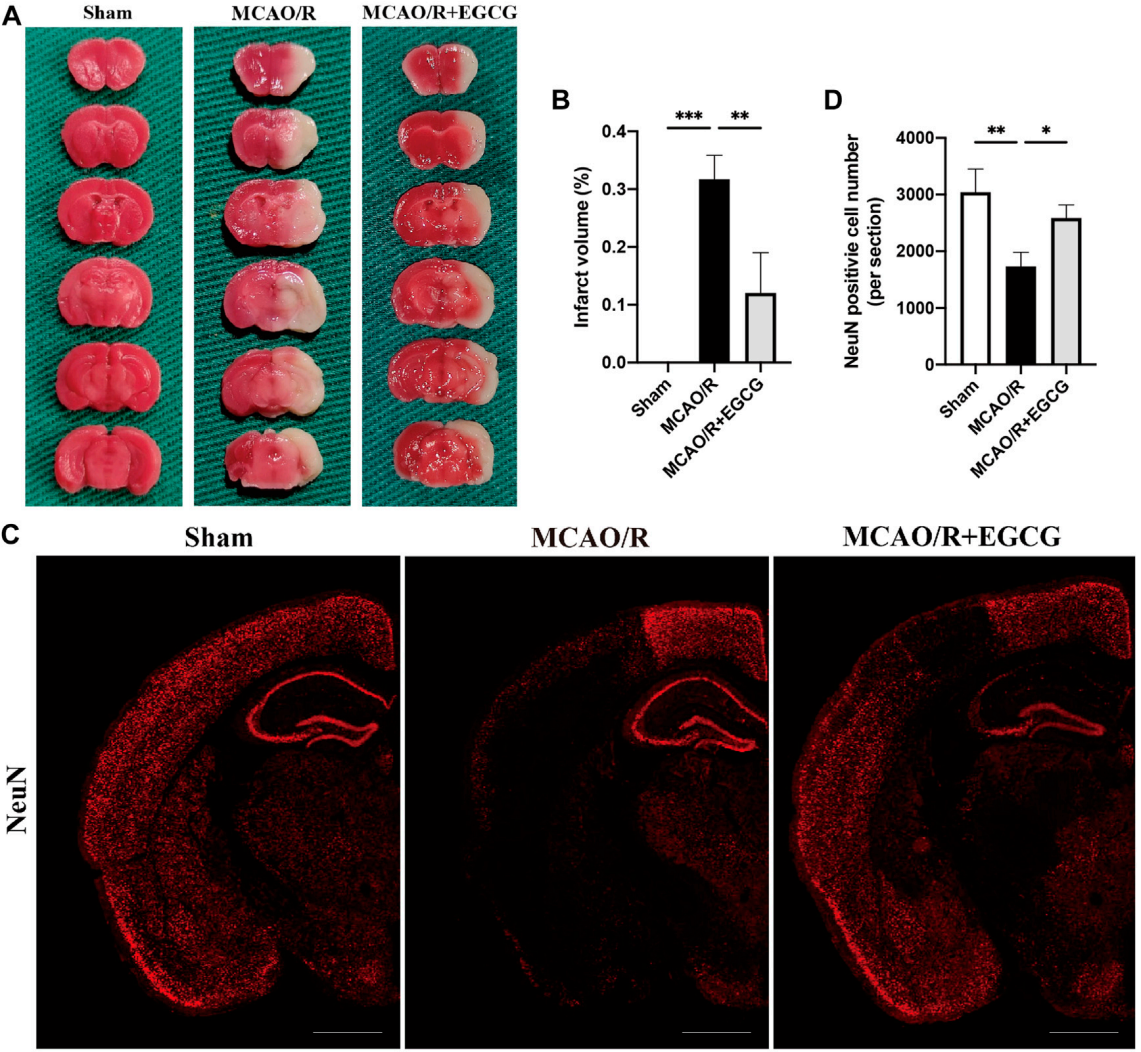
EGCG treatment mitigated cerebral ischemia/reperfusion injury (CIRI) in the mice model of middle cerebral artery occlusion/reperfusion (MCAO/R). **(A,B)** 2, 3, 5-Triphenyltetrazolium chloride (TTC) staining assay was performed to evaluate the infarct volume. **(C,D)** Immunofluorescent staining for NeuN. Data were shown in the semi-brain from the ipsilateral side of surgery on post-surgery day (PSD) 1 (scale bar = 1,000 μm). Each experimental datum was presented as mean ± standard deviation (*n* = 3 animals per group). *p < 0.05, **p < 0.01, ***p < 0.001 versus the specified group.

To determine the neuroprotective capacity of EGCG in stroke, TTC staining was conducted to evaluate the volume of infarction on PSD 1. TTC staining results showed that, compared with the sham group, the volume of infarction in the MCAO/R group was larger obviously, suggesting a severe brain ischemia injury, whereas administration with EGCG 1 h after reperfusion evidently decreased the infarct volume of MCAO/R mice (Figures 1A,B), indicating that treatment with EGCG relieved brain stroke injury in mice, which was similar to the previous research (Choi et al., 2004; Han et al., 2014; Zhang et al., 2015; Park et al., 2020).

In addition, to further verify the neuroprotective role of EGCG in stroke, immunofluorescent staining for the level of NeuN was conducted to evaluate neuron loss on PSD 1 (Wang H.-K. et al., 2021). As presented in Figures 1C,D, a semi-brain section screening was used, and the results showed that ischemia intrusion distinctly reduced the number of cells with immunoreactive NeuN, and this result was prevented by EGCG administration, indicating that EGCG treatment protected poststroke neuronal loss, which was consistent with the TTC results (Figures 1A,B). Thus, these data suggested that EGCG owned a neuroprotective potential in mice challenged with MCAO/R.

### EGCG mitigated CIRI through attenuating autophagy

**FIGURE 2:**
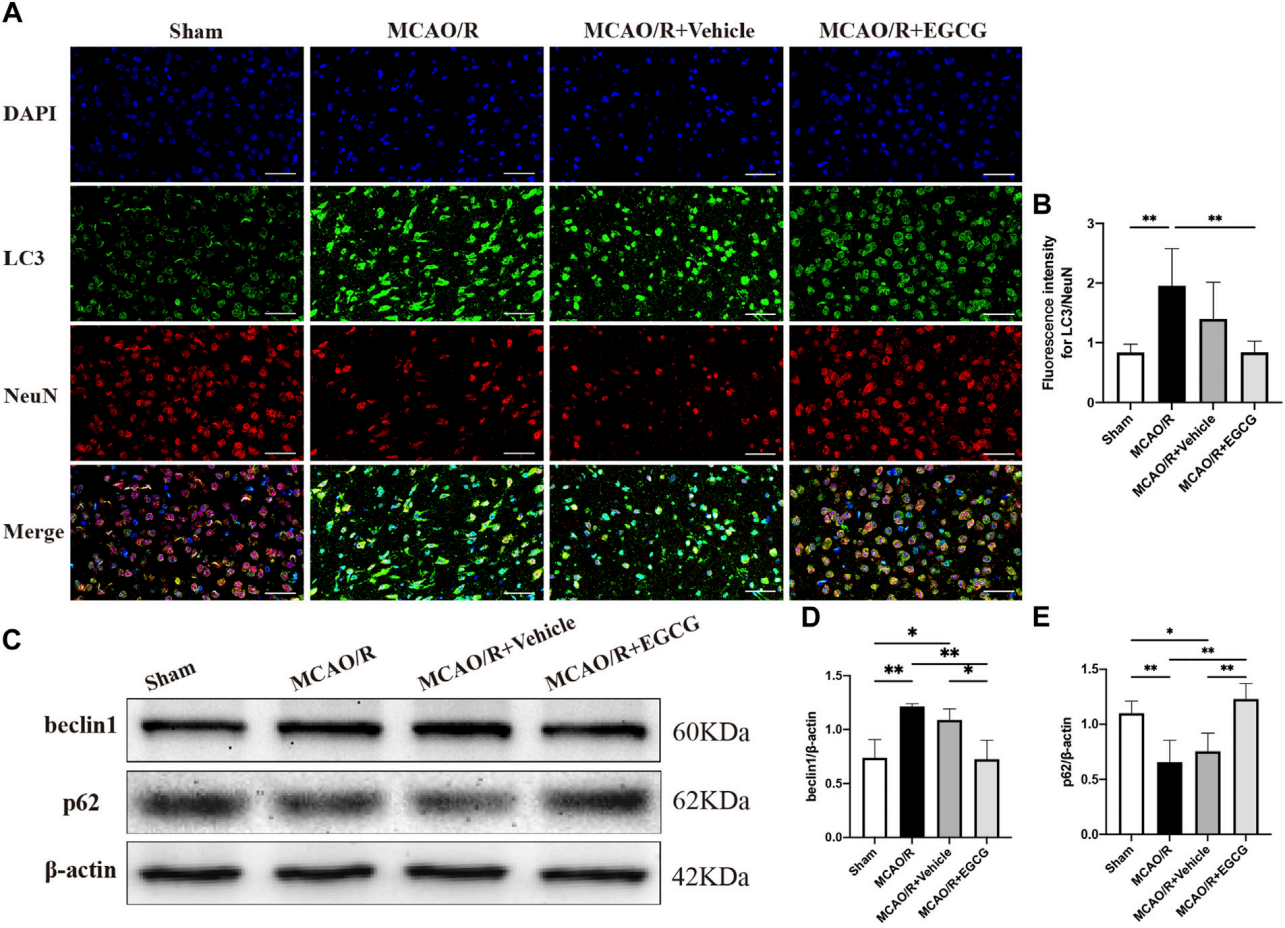
EGCG inhibited middle cerebral artery occlusion/reperfusion (MCAO/R)-induced autophagy. **(A,B)** The expression of LC3 was detected by immunofluorescent staining assay. Data were shown in the cortical penumbra area from the ipsilateral side of surgery on post-surgery day (PSD) 1 (scale bar = 20 μm). **(C)** The expression of beclin1 **(D)** and p62 **(E)** was assessed by western blotting assay. Each experimental datum was presented as mean ± standard deviation (*n* = 3 animals per group). *p < 0.05, **p < 0.01 versus the specified group.

After confirming the protective potential of EGCG in MCAO/R mice, the autophagic activity after stroke and the effect of EGCG on MCAO/R-induced autophagy were then examined by evaluating the expression of autophagy-associated proteins, LC3, beclin1, and p62. The expression of LC3, a specific marker for autophagy, was measured by immunofluorescent staining. As shown in Figures 2A,B, in comparison with the sham group, the expression of LC3 protein in the cortical penumbra area markedly increased after stroke, while EGCG administration considerably attenuated MCAO/R-induced increase in the LC3 level. Meanwhile, beclin1 and p62 protein levels were evaluated by Western blotting assay. As illustrated in Figures 2C–E, compared with the sham group, the expression level of beclin1 significantly increased on PSD 1, while the elevated expression of beclin1 induced by MCAO/R was prevented by EGCG treatment. In addition, the p62 level decreased after stroke, and the MCAO/R-induced down-regulated expression of p62 was also prevented by EGCG administration. The above results indicated that autophagy was activated by stroke, and EGCG was able to counteract the activation of poststroke autophagy to exert a neuroprotective effect.

### EGCG modulated the AKT/AMPK/mTOR phosphorylation pathway in CIRI

**FIGURE 3:**
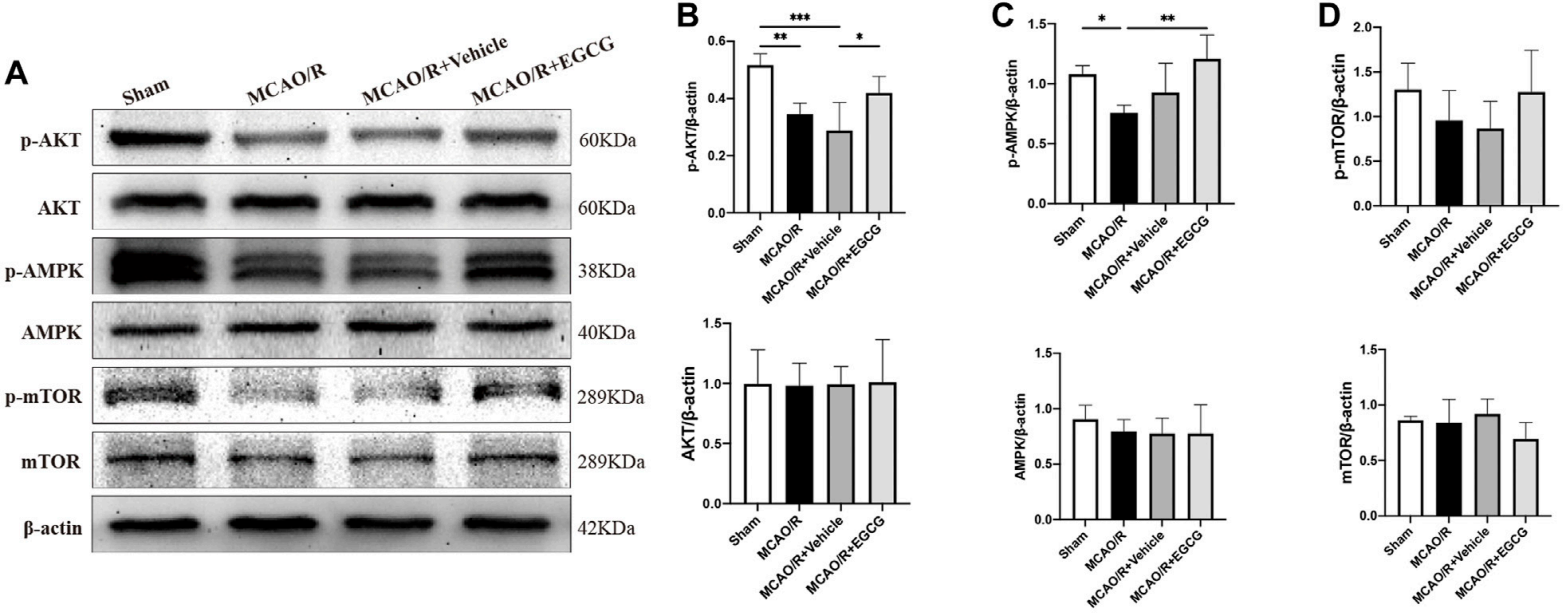
EGCG modulated the AKT/AMPK/mTOR phosphorylation pathway in middle cerebral artery occlusion/reperfusion (MCAO/R) mice. **(A)** The expression of p-AKT and AKT **(B)**, p-AMPK and AMPK **(C)**, p-mTOR and mTOR **(D)** was assessed by Western blotting assay. Each experimental datum was presented as mean ± standard deviation (*n* = 3 animals per group). *p < 0.05, **p < 0.01, ***p < 0.001 versus the specified group.

To explore the potential molecular mechanisms of EGCG in stroke, we determined the effect of EGCG on AKT, p-AKT, AMPK, p-AMPK, mTOR, and p-mTOR in the ischemic hemisphere brain tissue by Western blotting assay. As shown in Figure 3, in comparison with the sham group, the expression of p-AKT, p-AMPK, and p-mTOR was remarkably reduced on PSD 1, whereas treatment with EGCG prevented MCAO/R caused the reduced level of p-AKT, p-AMPK, and p-mTOR. And AKT, AMPK, and mTOR had no change. These findings suggested that the AKT/AMPK/mTOR phosphorylation pathway was involved in the underlying mechanisms of EGCG to exert a neuroprotective effect in stroke.

### GSK690693 and rapamycin reversed the effect of EGCG on ischemic brain tissue

**FIGURE 4:**
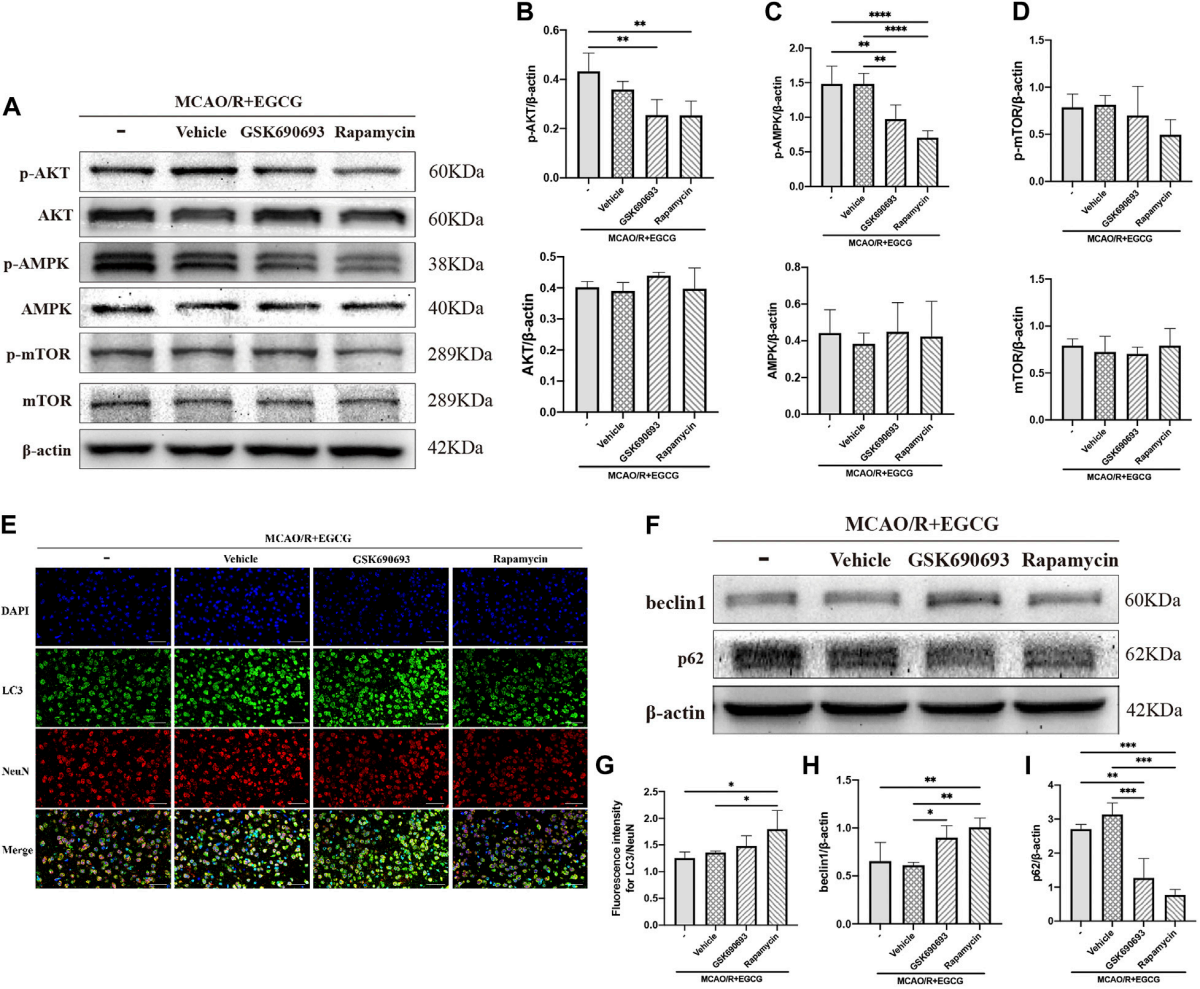
GSK690693 and rapamycin reversed the effect of EGCG in the ischemic brains of middle cerebral artery occlusion/reperfusion (MCAO/R) mice. **(A)** The expression of p-AKT and AKT **(B)**, p-AMPK and AMPK **(C)**, p-mTOR and mTOR **(D)** was assessed by Western blotting assay. **(E,G)** The expression of LC3 was detected by immunofluorescent staining assay. Data were shown in the cortical penumbra area from the ipsilateral side of surgery on post-surgery day (PSD) 1. **(F)** The expression of beclin1 **(H)** and p62 **(I)** was assessed by Western blotting assay (scale bar = 20 μm). Each experimental datum was presented as mean ± standard deviation (*n* = 3 animals per group). *p < 0.05, **p < 0.01, ***p < 0.001, ****p < 0.0001 versus the specified group.

GSK690693, an AKT/AMPK inhibitor (Rhodes et al., 2008; Levy et al., 2009; Altomare et al., 2010), and rapamycin, the inhibitor of mTOR (Edwards and Wandless, 2007), were used to further confirm that phosphorylated AKT, AMPK, and mTOR participated in the anti-autophagy effect of EGCG to protect against ischemic brain damage. As illustrated in Figures 4A–D and Supplementary Figure S1, administration with GSK690693 and rapamycin prevented EGCG-induced elevated levels of p-AKT, p-AMPK, and p-mTOR in MCAO/R mice. And AKT, AMPK, and mTOR had no change. These data verified the reversing effect of GSK690693 on p-AKT, p-AMPK, and rapamycin on p-mTOR in MCAO/R models applicated with EGCG.

After p-AKT, p-AMPK, and p-mTOR were blocked by inhibitors in MCAO/R mice treated with EGCG, autophagy-related proteins were measured. LC3 was measured by immunofluorescence staining. As presented in Figures 4E,G, the EGCG treatment-induced down-regulated expression of LC3 level in MCAO/R mice was prevented by rapamycin and GSK690693 precondition. In addition, beclin1 and p62 were measured by Western blotting assay. As illustrated in Figures 4F,H,I and Supplementary Figure S1, both GSK690693 and rapamycin precondition were able to prevent the EGCG-induced decreased expression of beclin1 and the increased expression of p62 level in MCAO/R models. Accordingly, these data favored the point that autophagy-related proteins could be influenced after the AKT/AMPK/mTOR phosphorylation pathway was inhibited, further indicating that EGCG inhibited MCAO/R-induced autophagy in the AKT/AMPK/mTOR phosphorylation-dependent manner.

### EGCG protected HT22 cells from OGD/R-challenged damage

**FIGURE 5:**
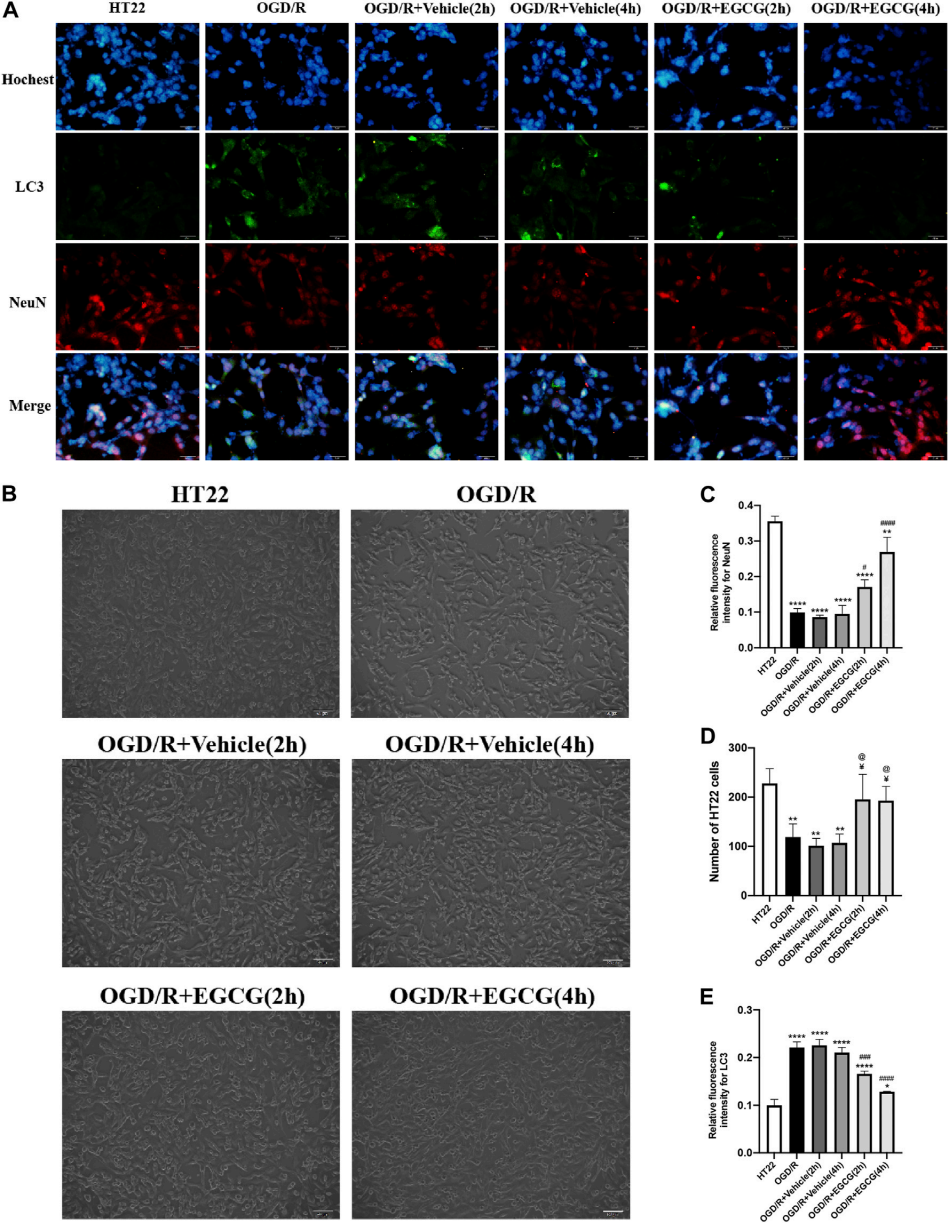
EGCG protected HT22 cells from oxygen-glucose deprivation/reoxygenation (OGD/R) damage. **(A)** Immunofluorescent staining for NeuN **(C)** and LC3 **(E)** (scale bar = 50 μm). **(B,D)** The number of HT22 cells in different groups. Each experimental datum was presented as mean ± standard deviation (*n* = 3 per group). *p < 0.05, **p < 0.01, ****p < 0.0001 versus HT22 group, #*p* < 0.05, ###*p* < 0.001, ####*p* < 0.0001 versus OGD/R group, ¥ *p* < 0.05 versus OGD/R + Vehicle (2 h) group, @*p* < 0.05 versus OGD/R + vehicle (4 h) group.

After OGD/R invasion, immunofluorescent staining for NeuN was examined to confirm the protective role of EGCG on HT22 cells challenged by OGD/R. As presented in Figures 5A,C, the OGD/R challenge markedly reduced the number of HT22 cells with immunoreactive NeuN, but this effect was prevented by EGCG treatment, and the number of HT22 cells with immunoreactive NeuN was more in 4 h than in 2 h of EGCG incubation. Figures 5B,D show the cell pictures in different groups, and the number of HT22 cells was assessed by ImageJ software. And Figures 5B,D also support the protective effect of EGCG on HT22 cells, which is in accordance with Figures 5A,C. Thus, these results supported the ability of EGCG in protecting HT22 cells from the OGD/R challenge, which was consistent with the results *in vivo* (Figures 1C,D), and the efficacy of EGCG might be positively related to the time of incubation.

### EGCG suppressed OGD/R-induced autophagy to protect HT22 cells

**FIGURE 6:**
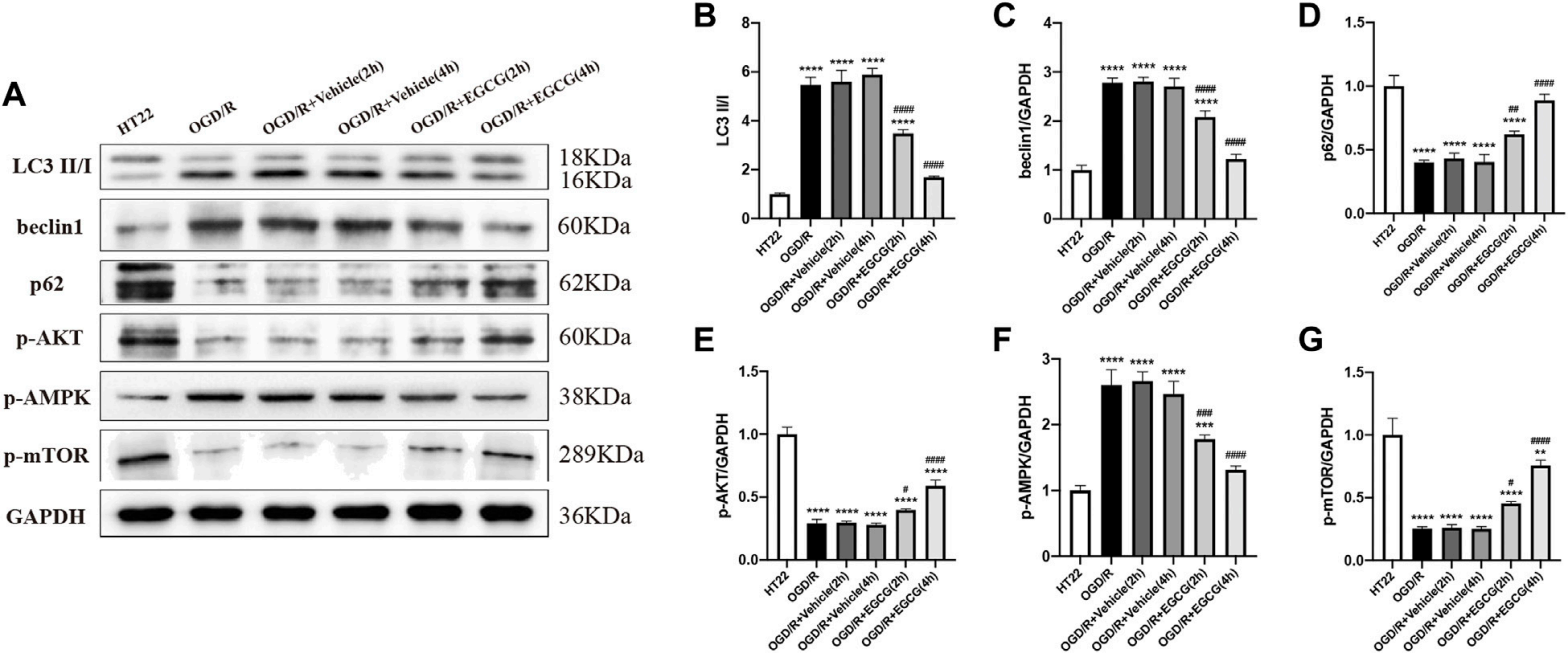
EGCG suppressed autophagy and modulated the AKT/AMPK/mTOR phosphorylation pathway in oxygen-glucose deprivation/reperfusion (OGD/R)-challenged HT22 cells. **(A)** The expression of LC3 II/I **(B)**, beclin1 **(C)**, p62 **(D)**, p-AKT **(E)**, p-AMPK **(F)**, and p-mTOR **(G)** was assessed by Western blotting assay. Each experimental datum was presented as mean ± standard deviation (*n* = 3 per group). **p < 0.01, ***p < 0.001, ****p < 0.0001 versus HT22 group, #*p* < 0.05, ##*p* < 0.01, ###*p* < 0.001, ####*p* < 0.0001 versus OGD/R group.

Autophagy-related proteins were examined to investigate the autophagic activity after OGD/R and the role of EGCG on OGD/R-induced autophagy in HT22 cells. As shown in Figures 5A,E, 6A–D, the LC3 level and beclin1 level increased and p62 level decreased after OGD/R, suggesting that OGD/R led to autophagic activation in HT22 cells. In addition, the OGD/R invasion-induced up-regulated expression of LC3 and beclin1 and down-regulated expression of p62 were prevented by EGCG treatment, which was in accordance with the results *in vivo* (Figures 2A–E). Moreover, the preventing effects of EGCG on these autophagic proteins were more evident in 4 h than in 2 h of incubation time. Accordingly, these results suggested that EGCG substantially inhibited the autophagic activity to protect HT22 cells against OGD/R damage.

### EGCG regulated the AKT/AMPK/mTOR phosphorylation pathway in OGD/R-challenged HT22 cells

To confirm the molecular mechanisms by which EGCG exerted a protective effect on OGD/R-damaged HT22 cells, p-AKT, p-AMPK, and p-mTOR were determined by Western blotting assay. As presented in Figures 6A,E–G, the expressions of p-AKT level and p-mTOR level were remarkably reduced after OGD/R, and treatment with EGCG prevented the OGD/R-induced down-regulated expression of p-AKT and p-mTOR, which were consistent with animal results (Figures 3A,B,D). In comparison, after OGD/R invasion, p-AMPK was considerably increased, and EGCG treatment prevented the elevated expression of p-AMPK induced by OGD/R, which was inconsistent with animal results (Figures 3A,C). And the preventing effects of EGCG on these proteins of the pathway were more evident in 4 h than in 2 h of incubation time. Therefore, the above data indicated that the underlying mechanisms by which EGCG protected HT22 cells from OGD/R were related to the AKT/AMPK/mTOR phosphorylation pathway.

### GSK690693 and rapamycin reversed the effect of EGCG on OGD/R-challenged HT22 cells

**FIGURE 7:**
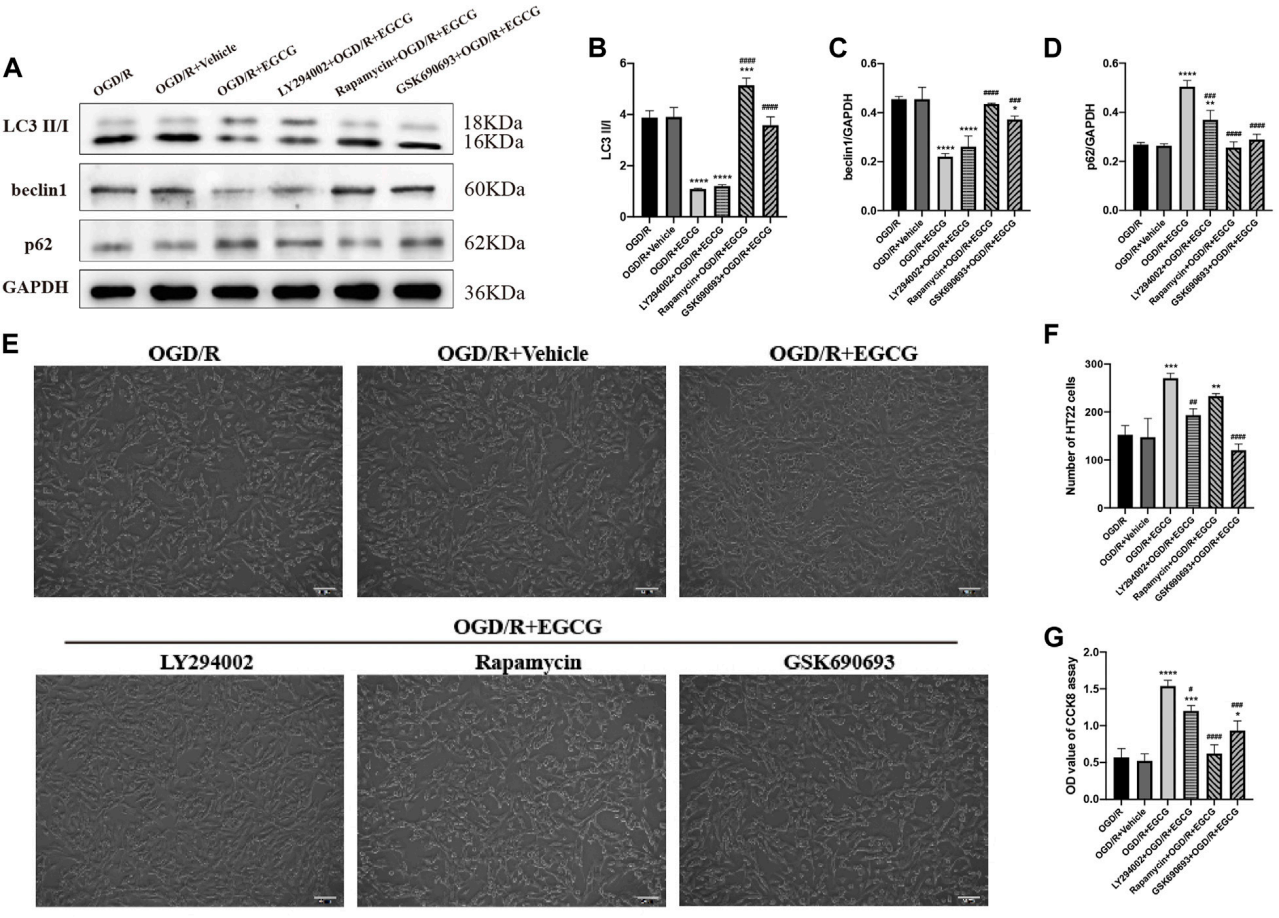
LY294002, GSK690693, and rapamycin reversed the effect of EGCG in HT22 cells challenged by oxygen-glucose deprivation/reperfusion (OGD/R). **(A)** The expression of LC3 II/I **(B)**, beclin1 **(C)**, and p62 **(D)** was assessed by Western blotting assay. **(E)** The number **(F)** and cell viability **(G)** of HT22 cells in different groups. Each experimental datum was presented as mean ± standard deviation (*n* = 3 per group). *p < 0.05, **p < 0.01, ***p < 0.001, ****p < 0.0001 versus OGD/R group, #*p* < 0.05, ##*p* < 0.01, ###*p* < 0.001, ####*p* < 0.0001 versus OGD/R + EGCG group.

To further confirm that the phosphorylated AKT, AMPK, and mTOR were required for the anti-autophagy effect of EGCG to exert the protective effect in OGD/R models, we added LY294002, GSK690693, and rapamycin to OGD/R-challenged HT22 cells, respectively, followed by treatment with EGCG. Autophagy-associated proteins were then examined by Western blotting assay. As shown in Figures 7A–D, the EGCG treatment-induced down-regulated expression of LC3 and beclin1 and the up-regulated expression of p62 in OGD/R models were prevented by GSK690693 and rapamycin precondition. In addition, the LY294002 precondition was able to prevent the EGCG treatment-induced increased expression of p62 in OGD/R models, while the effect of LY294002 on LC3 and beclin1 was not statistically significant. Figures 7E–G also show that the protective effect of EGCG on OGD/R-challenged HT22 cells can be weakened by LY294002, GSK690693, and rapamycin precondition. Accordingly, these data suggested that autophagy-related proteins could be influenced after the AKT/AMPK/mTOR phosphorylation pathway was inhibited, which was in accordance with animal experiments (Figures 4E–I), further indicating that EGCG inhibited the OGD/R-induced autophagy of HT22 cells in an AKT/AMPK/mTOR phosphorylation-dependent manner.

## Discussion

Stroke is largely related to disability and death all around the world. Neuron destruction is usually found in stroke, involving autophagy. Herein, we examined the role of EGCG on ischemic injury in MCAO/R models of mice and OGD/R models of HT22 cells. The results supported the point that EGCG protected neurons against ischemic injury both *in vivo* and *in vitro*. Furthermore, the neuroprotective effect of EGCG to ameliorate stroke was associated with the suppression of autophagic activation. In terms of molecular mechanisms, EGCG modulated autophagic activity via the AKT/AMPK/mTOR phosphorylation pathway. In summary, EGCG exerted a neuroprotective effect by suppressing autophagy in an AKT/AMPK/mTOR phosphorylation-dependent manner.

Autophagy is a highly regulated process, participating in multiple pathophysiological processes of many diseases (Wirawan et al., 2012). Mounting evidence has suggested that autophagy activation participated in ischemic stroke closely and exerted divergent roles in stroke’s pathological and physiological changes (Sheng and Qin, 2015). The research by Sun et al. (2020b) indicated that eugenol, an active ingredient extracted from traditional herbal medicine, played a neuroprotective role by the enhancement of autophagy flux. Besides, Ginkgo biloba leaf extract (EGb-761), also the extraction of a traditional Chinese herb, was reported to elicit neuroprotection against ischemic brain injury through enhancing autophagy (Yihao et al., 2021). The above studies suggested that enhancing autophagy activation exerted a protective role on the ischemic injury. However, another voice cannot be ignored for the harmful role of autophagy activation in CIRI. Wang L. et al. (2020) demonstrated that Tanshinone IIA (TSA), the major component extracted from traditional medicine, protected brain tissues from ischemic injury by suppressing autophagy. The research by Zhang et al. (2020b) showed that deltonin, an effective ingredient obtained from a type of Chinese medicine, reduced autophagy activity to play a beneficial role in brain stroke. These studies supported the point that the reduction of autophagy activity was beneficial for stroke. Different types of animals, different disease models, and intensity and duration time of ischemia were possibly the reasons for the different roles of autophagy on CIRI (Zhang et al., 2019; Sun et al., 2020b). In the present study, we revealed that autophagy activation was deleterious in the acute phase of CIS, and EGCG mitigated CIRI via inhibition of autophagy activation, given the evidence that EGCG postcondition could suppress autophagic activity and further reduce the volume of infarction and protect poststroke neuronal loss.

Multiple autophagy-related proteins are involved in the process of autophagy. Beclin1, a central component in the autophagy complex, can bind to ligands and thereby initiate autophagosome formation and play a central role in the process of the movement of autophagy-associated proteins to a pre-autophagosome structure (Kang et al., 2011; Wu et al., 2017). During autophagy, LC3 is cleaved into LC3 I by autophagy-related (Atg) genes four proteases and then connected with phosphatidylethanolamine (PE) to produce LC3 II through the activation of Atg7, Atg3, and Atg12 complex in order. And LC3 II exerts a central role in the biogenesis/maturation of autophagosome membrane. Thus, LC3 II is associated with the amount of the formation of autophagosomes, and LC3 II is served as the biological marker to detect autophagy (Lee and Lee, 2016). P62, a cargo protein of ubiquitination substrates, is reported to participate in the degradation process of autophagy (Jiang et al., 2015). It can combine with LC3 directly and be selectively delivered into autophagosomes, and the amount of p62 within the cell is negatively correlated with the intensity of autophagy (Hou et al., 2021). In brief, when autophagy occurs, beclin1 is required for the construction of autophagosomes, and LC3 is cleaved into LC3 I and LC3 II and combines into autophagosomes simultaneously. And p62 is one of the proteins that are sequestered and degraded by the process of autophagy to supply energy to retain metabolic balance within cells. According to our results, EGCG treatment prevented ischemia and reperfusion invasion-mediated upregulation of the expression of beclin1 and LC3 and downregulation of the expression of p62, in both MCAO/R models of mice and OGD/R models of HT22 cells, indicating that the suppression of poststroke autophagy activity was related to the role of EGCG treatment to ameliorate ischemic brain injury.

Thereafter, the potential molecular mechanisms by which EGCG modulated autophagy were then explored. So far, the mammalian target of rapamycin (mTOR) is believed to be the main regulator of autophagy in the mammalian system (Abdul et al., 2020). It is reported that mTOR is critical for autophagosome formation and maturation, and its inactivation is required for the process of autophagy (Hou et al., 2021). A multitude of signals is integrated into the mTOR pathway. AKT is the upstream of mTOR and modulates mTOR activation (Heras-Sandoval et al., 2014). AMP-activated protein kinase (AMPK) plays an important role in keeping the balance of metabolic processes and is reported to be linked with the regulation of autophagy (Heras-Sandoval et al., 2014). Some studies suggested that ischemia postcondition mitigated ischemic stroke by suppressing autophagy *via* promoting the phosphorylation of AKT and mTOR (Yang et al., 2019; Wang H. et al., 2020; Wang M. M. et al., 2020; Meng et al., 2021), while others revealed that the poststroke treatment ameliorated CIRI through inhibiting autophagy via suppressing the phosphorylation of AKT, AMPK, and mTOR (Wang J. F. et al., 2018; Zhang et al., 2020a; Ma et al., 2020; Liu N. et al., 2021; Yang et al., 2021; Zhang et al., 2022). Therefore, the interaction between the phosphorylation of AKT, AMPK, and mTOR and poststroke treatment-induced autophagy suppression is complicated and remains to be further elucidated. In the present study, in MCAO/R models of mice, we revealed that promoting the phosphorylation of AKT, AMPK, and mTOR was involved in the protective mechanisms of EGCG administration. Moreover, after the phosphorylation of AKT, AMPK, and mTOR was blocked by GSK690693 and rapamycin, we found that EGCG treatment-induced autophagy suppression could be prevented. In addition, we obtained similar results in OGD/R models of HT22 cells. EGCG treatment could inactivate autophagy and promote the phosphorylation of AKT and mTOR while inhibiting the phosphorylation of AMPK. And the inhibition of autophagy caused by EGCG administration could be prevented by the blocking effect of GSK690693 and rapamycin on the phosphorylation of AKT, AMPK, and mTOR. The difference in the phosphorylated AMPK in MCAO/R mice and OGD/R-challenged HT22 cells might be contributed to different pathophysiological environments *in vivo* and *in vitro*. Collectively, these results indicated that EGCG inhibited ischemia-induced autophagy in an AKT/AMPK/mTOR phosphorylation-dependent manner to exert a neuroprotective effect.

Certainly, there remain some limitations in this article. Aging is the prime culprit for most neurodegenerative events (Uddin et al., 2020), including stroke; thus, modeling with older mice may be a better option. Besides, glial and endothelial cells are also important parts of brain tissues, whether the protective effect of EGCG on glial and endothelial cells is similar to that of neurons remains to be further verified. In addition, other types of cell death—necrosis, necroptosis, and apoptosis—do exist in the pathological process of stroke, the effect and mechanisms of EGCG on these ways of cell death, and the crosstalk between these ways of cell death are waiting to be elucidated. Of course, EGCG can never replace tPA as a specific drug, but it can become an indispensable adjuvant drug for the treatment of stroke. Thus, more precise mechanisms of the curative effect of EGCG in stroke still need to be improved to provide a solid foundation for its clinical application in the future.

## Conclusion

Taken together, our results indicated that the neuroprotective role of EGCG against CIRI was associated with the suppression of autophagy through the AKT/AMPK/mTOR phosphorylation pathway. The findings provide new insights into the potential mechanisms of the role of EGCG on autophagy and cerebral ischemic injury and may help design therapeutic strategies with more efficacy for stroke.

## Data Availability

The original contributions presented in the study are included in the article/Supplementary Material; further inquiries can be directed to the corresponding authors.
