# Supplementary material for: EGCG protects the mouse brain against cerebral ischemia/reperfusion injury by suppressing autophagy via the AKT/AMPK/mTOR phosphorylation pathway
Source: Front Pharmacol. 2022 Sep 6;13:921394. doi: 10.3389/fphar.2022.921394 (PMC9489224; doi:10.3389/fphar.2022.921394)
Supplement: Supplementary file 1 [file DataSheet1.docx]

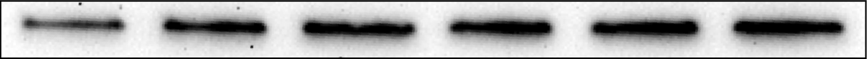

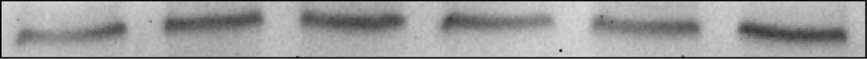

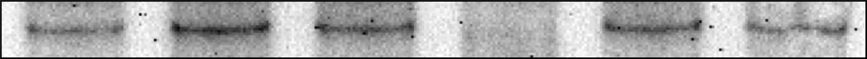

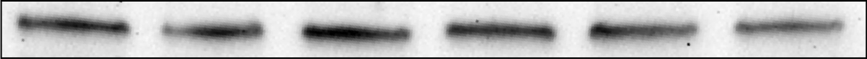

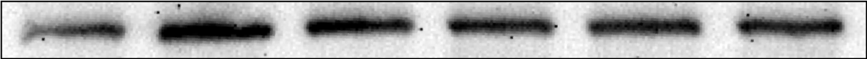

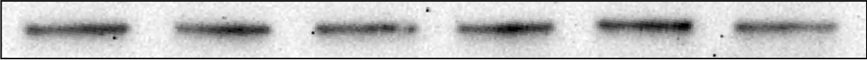

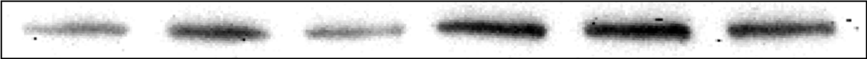

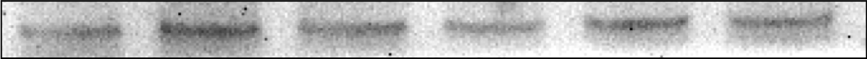


**Figure S1** EGCG inhibit autophagy via AKT/AMPK/mTOR phosphorylation pathway. The expression of p62, p-AKT, AKT, p-AMPK, AMPK, p-mTOR, mTOR were assessed by western blotting assay.

GSK690693+MCAO/R+EGCG

Rapamycin+MCAO/R+EGCG

Rapamycin+MCAO/R

GSK690693+MCAO/R

MCAO/R+EGCG

MCAO/R

42KDa

289KDa

289KDa

40KDa

38KDa

60KDa

60KDa

62KDa

β-actin

mTOR

p-mTOR

p-AMPK

AMPK

AKT

p-AKT

p62
